# Peripapillary Intrachoroidal Cavitations. The Beijing Eye Study

**DOI:** 10.1371/journal.pone.0078743

**Published:** 2013-10-24

**Authors:** Qi Sheng You, Xiao Yan Peng, Chang Xi Chen, Liang Xu, Jost B. Jonas

**Affiliations:** 1 Beijing Institute of Ophthalmology, Beijing Tongren Eye Center, Beijing Tongren Hospital, Capital Medical University, Beijing Ophthalmology and Visual Science Key Laboratory, Beijing, China; 2 Department of Ophthalmology, Medical Faculty Mannheim of the Ruprecht-Karls-University of Heidelberg, Mannheim, Germany; Dalhousie University, Canada

## Abstract

**Purpose:**

To assess prevalence, size and location of peripapillary intrachoroidal cavitations (PICCs) and their associations in a population-based sample. .

**Methods:**

The population-based Beijing Eye Study 2011 included 3468 individuals with a mean age of 64.6±9.8 years (range:50-93 years). A detailed ophthalmic examination included enhanced depth imaging of the choroid by spectral-domain optical coherence tomography and fundus photography. PICCs were defined as triangular thickening of the choroid with the base at the optic disc border and a distance between Bruch´s membrane and sclera of ≥200μm. Parapapillary large choroidal vessels were excluded.

**Results:**

Out of 94 subjects with high myopia (refractive error <-6.0 diopters or axial length >26.5mm in right eyes), OCT images were available for 89 (94.7%) participants. A PICC was detected in 15 out of these 89 highly myopic subjects (prevalence:16.9±4.0%) and in none of hyperopic, emmetropic or medium myopic subgroups each consisting of 100 randomly selected subjects. Mean PICC width was 4.2±2.3 hours (30°) of disc circumference and mean length was 1363±384μm. PICCs were located most frequently (40%) at the inferior disc border. On fundus photos, a typical yellow-orange lesion was found in 8 (53%) eyes with PICCs. In binary regression analysis, presence of PICCs was significantly associated with optic disc tilting (*P*=0.04) and presence of posterior staphylomata (P=0.046).

**Conclusions:**

Prevalence of PICCs in the adult Chinese population was 16.9±4.0% in the highly myopic group, with no PICCs detected in non-highly myopic eyes. PICCs were located most frequently at the inferior optic disc border. Only half of the PICCs detected on OCT images showed a yellow-orange lesion on fundus photos. Presence of PICC was significantly associated only with an increased optic disc tilting and presence of posterior staphylomata, while it was not associated with axial length, refractive error or other ocular or systemic parameters.

## Introduction

The peripapillary intrachoroidal cavitation (PICC) was ophthalmoscopically described by Freund and colleagues in 2003 as an orange-yellow lesion located in the peripapillary region usually at the inferior border of a myopic peripapillary conus [1]. It was initially named “peripapillary detachment in pathologic myopia”. After the introduction of optical coherence tomography (OCT) into clinical practice, one observed that PICC was characterized by an intrachoroidal hyporeflective space with normal overlying retina and retinal pigment epithelium (RPE) [2-10]. It was additionally detected that eyes with PICC tended to show glaucoma-like visual field defects [11]. Recent studies applying swept-source OCT and the enhanced depth imaging mode of spectral-domain OCT demonstrated a posterior deformation of the sclera in regions with PICC [8-10,12]. Although the pathogenesis and clinical significance of PICC has remained unclear, it is important to recognize PICC as a morphologic entity in highly myopic eyes, different from other fundus lesions such as peripapillary choroidal neovascularization or amelanotic choroidal tumors. In hospital-based clinical case series studies, the prevalence of PICC in highly myopic eyes ranged between 4.9% and 11.0% [4,5]. Since hospital-based investigations inherently carry the risk of a bias by a selection artifact due to the referral of the patients, we conducted this study to assess the prevalence of PICC in a population-based group of subjects and to examine the factors associated with the presence of PICCs. 

## Methods

### Ethics Statement

The Medical Ethics Committee of the Beijing Tongren Hospital approved the study protocol and all participants gave informed written consent. 

The Beijing Eye Study 2011 is a population-based cross-sectional study in Northern China as described in detail previously [13,14]. The details of the study have been described in detail previously. The only eligibility criterion for inclusion into the study was an age of 50+ years. Out of 4403 eligible individuals, 3468 (78.8%) subjects (1963 (56.6%) women) participated. The mean age was 64.6 ± 9.8 years (range, 50 - 93 years). All study participants underwent an interview with standardized questions on their family status, level of education, physical activity, and known major systemic diseases; anthropometric parameters such as body height and weight were measured; blood pressure was measured, and fasting blood samples were examined for the concentration of blood lipids, glucose, glycosylated hemoglobin HbA1c, creatinine, and C-reactive protein. The ophthalmic examination included measurement of presenting visual acuity, uncorrected visual acuity, and best corrected visual acuity, tonometry, refractometry, slit lamp assisted examination of the anterior and posterior segment of the eye, and digital photography of the cornea, lens, macula and optic disc (fundus camera Type CR6-45NM; Canon Inc., Tokyo, Japan). Biometry for measurement of the anterior corneal curvature, central corneal thickness, anterior chamber depth, lens thickness and axial length (optical low-coherence reflectometry; Lensstar 900® Optical Biometer, Haag-Streit, 3098 Koeniz, Switzerland) was carried out in the right eyes of the study participants. Using fundus photographs, we assessed also the presence of retinal vein occlusions, diabetic retinopathy, and glaucomatous optic neuropathy, as defined and described in detail previously [13,14]. 

Spectral-domain optical coherence tomography (Spectralis®, Wavelength: 870nm; Heidelberg Engineering Co., Heidelberg, Germany) with the enhanced depth imaging (EDI-OCT) mode was performed after pupil dilation for right eyes [8,15]. Six radial line B-scans centered on optic disc center of the right eye of each participant were performed to obtain the tomographic images of the peripapillary region. Thirty-one sections covered a 30° x 30° large rectangle centered onto the fovea were obtained. All the images were taken by an experienced technician (CXC) and the images were examined by a retinal specialist (XYP) and retinal fellow (QSY). In case of doubt, the images were re-examined by a panel of ophthalmologists (QSY, JBJ, XYP and LX). The OCT images were analyzed with Heidelberg software (Heidelberg Viewer Module 5.3a). 

**Figure 1:**
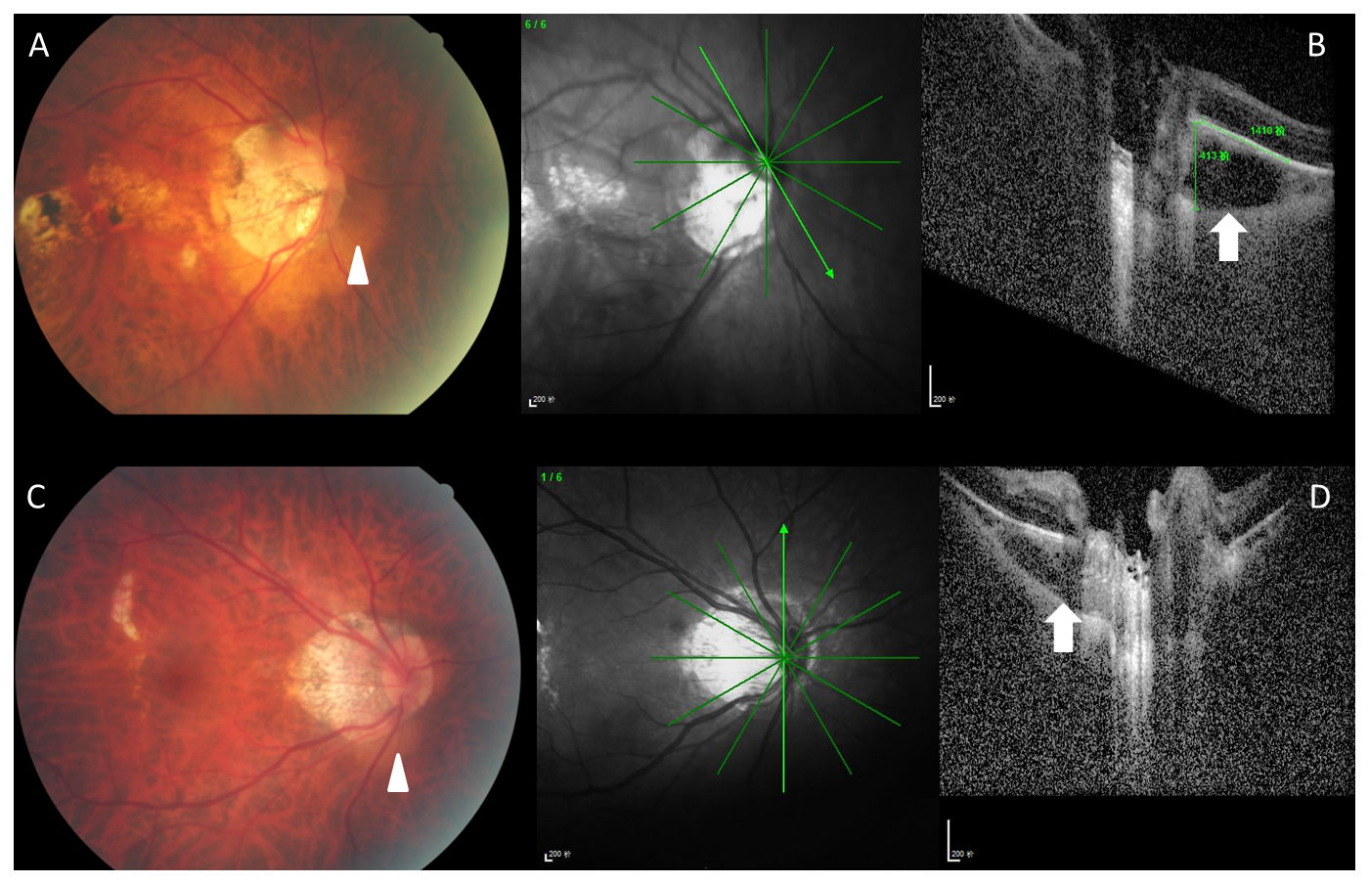
Color fundus photographs (Figure 1A, C) and corresponding optical coherence tomograms (Figure 1B, D) of highly myopic patients with a peripapillary intrachoroidal cavitation (PICC). Note: triangular thickening of the choroid (white arrow in Figures B, D) with the base at the optic disc border; corresponding yellow-orange lesions (white arrow heads) on fundus photographs (Figure 1 A, C).

**Figure 2:**
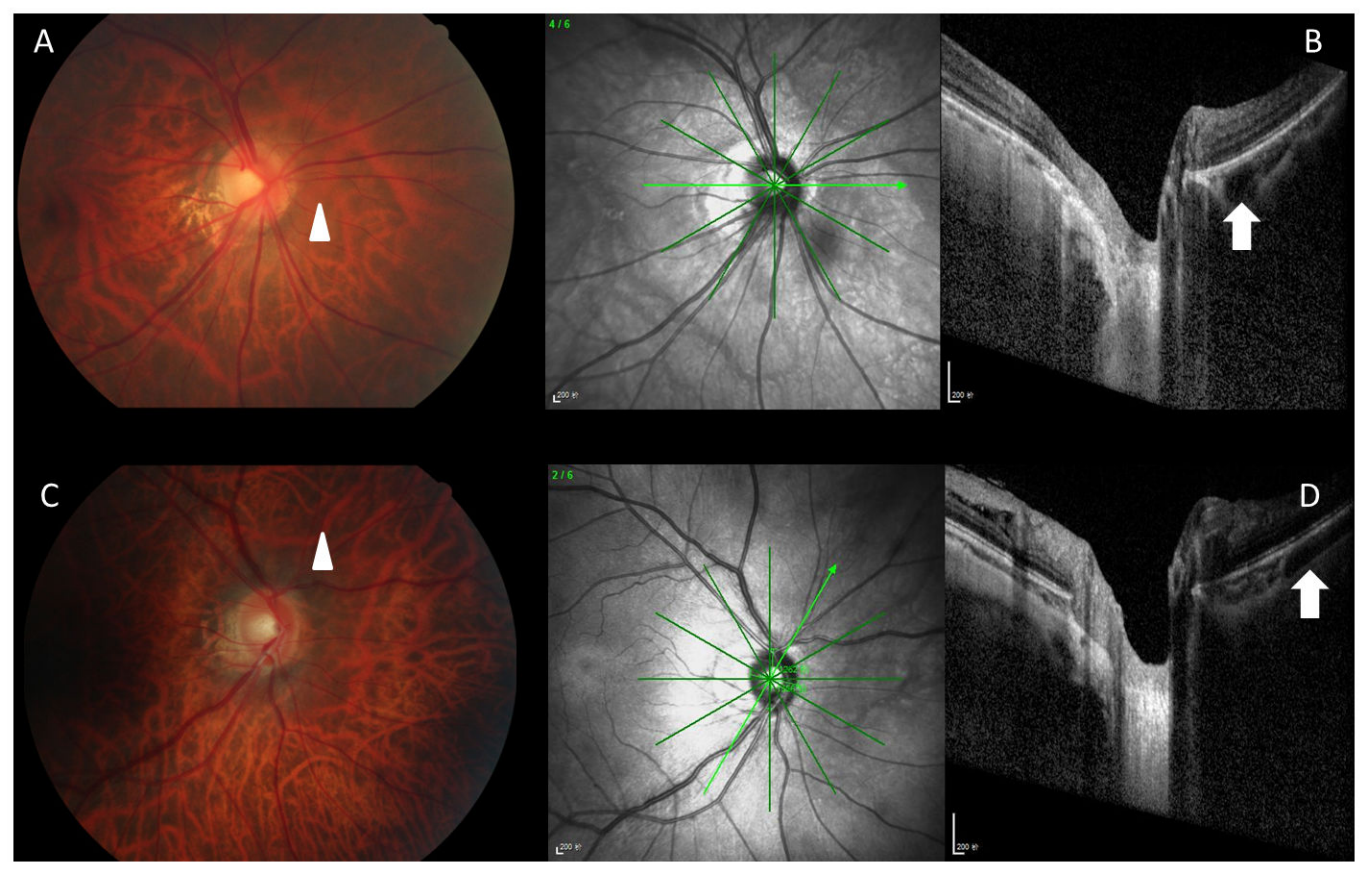
Color fundus photographs (Figure 2A, C) and corresponding optical coherence tomograms (Figure 2B, D) of patients with a pseudo-PICC (peripapillary intrachoroidal cavitation). The round structure (Figure 2B, white arrow) and the tube-like structure (Figure 2D; white arrow) with low reflection on the optical coherence tomograms correspond to large choroidal vessels on the fundus photographs (Figure 2A. C; white arrow heads) and were not considered to be a PICC.

The tomographic images of peripapillary and macular region of the right eye of all participants with high myopia (defined as a refractive error of ≤-6 diopters or an axial length of ≥26.5 mm) and of a randomly selected group of 100 participants with emmetropia (defined as a refractive error >-1 diopter and < +1diopter), 100 participants with hyperopia (defined as a refractive error ≥+1 diopter), and 100 participants with low myopia (defined as a refractive error >-6 diopters and ≤-1 diopter) were analyzed for PICC. PICCs were defined as a triangular thickening of the choroid with the base at the optic disc border and a distance between Bruch´s membrane and sclera of ≥200μm (Figure 1). A tube-like or round area with low reflection on the OCT images corresponding to a choroidal vessel on the fundus photographs was considered to be a large choroidal vessel (Figure 2). The width of the PICC was measured and expressed in twelfth parts of the optic disc circumference (“hours”). The length of the cavitation was measured in micrometer (Figure 1 B). The center location of the lesion was recorded as superior, superior nasal, nasal, nasal inferior, inferior, inferior temporal, temporal and temporal superior. The orientation of the longest axis of the optic disc was assessed using the same grading. The shape of the optic disc was additionally assessed by calculating the ratio shortest optic disc diameter to the longest disc diameter. The ratio was called the “optic disc tilt index”. As imaged by the OCT, the parapapillary region was differentiated into alpha zone, beta zone and gamma zone. Alpha zone was defined as presence of Bruch´s membrane with irregular RPE; beta zone was characterized by the presence of Bruch´s membrane without RPE; and gamma zone was defined by the absence of Bruch´s membrane [16,17]. As descried recently, the OCT images of the macular regions were searched for epiretinal membranes, photoreceptor inner segment / outer segment disruption, macular holes, defects in the RPE / Bruch’s membrane complex, RPE clumping or choroidal neovascularization [18]. Examining the fundus photographs, the typically yellow-orange ophthalmoscopical appearance lesion of PICCs (Figure 1 A, C.) was recorded. Other lesions typical for myopic retinopathy such as myopic conus, lacker cracks, chorioretinal atrophy and Fuchs’ spot were additionally assessed [19]. 

Statistical analysis was performed using a commercially available statistical software package (SPSS for Windows, version 21.0, IBM-SPSS, Chicago, IL). In a first step, we examined the prevalence (presented as mean ± standard error) of PICC. The mean values of other parameters were expressed as mean ± standard deviation. In a second step, we examined associations between the prevalence of PICC and other ocular and systemic parameters in univariate analysis. In a third step, we performed a binary regression analysis, with the presence of a PICC as dependent parameter and those parameters as independent parameters which were significantly associated with the PICC in univariate analysis. From the list of independent parameters we dropped step by step those with the highest P-value, until eventually all independent parameters showed a statistically significant association with the presence of PICC. Odds ratios (OR) and 95% confidence intervals (CI) were presented. All *P*-values were 2-sided and were considered statistically significant when the values were less than 0.05. 

## Results

Out of the 3468 subjects, high myopia defined as a refractive error (spherical equivalent) of less than -6.0 diopters or an axial length longer than 26.5 mm in the right eye was present in 94 (2.7±0.3%) participants (39 men). Among them, the OCT images of 89 (94.7%) participants were available and evaluable for the peripapillary region. For 5 (5.3%) eyes, OCT images either had not been taken or the images could not be examined due to vitreous clouding or cataract. The mean age of the highly myopic participants was 64.6 ± 8.7 years (range: 50 - 83 years), which was significantly (*P*=0.02) younger than the mean age of the hyperopic group (67.8 ± 8.6 years), but not significantly (*P*=0.18) different from the age of the low myopic group (66.4 ± 10.5 years) nor the age of the emmetropic group (62.4 ± 9.1 years; *P*=0.12). The demographic characteristics of the participants were shown in Table 1. 

**Table 1:** The demographic characteristics (Mean ± Standard Deviation; Range) of the study participants Stratified by Refraction Error (Spherical Equivalent (SE)).

Groups	Age (Years)	Men / Women	Region of Habitation (Rural/Urban)
High Myopia (SE ≤- 6 Diopters or Axial Length ≥26.5 mm)	64.6 ± 8.7 (50 - 83)	39/50	30/59
Low myopia (-6 Diopter < SE <-1 Diopter)	66.4 ± 10.5 (50 - 87)	39/56	31/64
Emmetropia (-1 Diopter ≤ SE ≤+1 Diopter)	62.4 ± 9.1 (50 - 88)	46/52	58/40
Hyperopia (SE > +1 Diopter	67.8 ± 8.6 (52 - 89)	41/55	37/59

A PICC was detected in 15 out of 89 participants of the highly myopic group (prevalence: 16.9 ± 4.0%), while none of the subjects of the other groups showed a PICC. The mean size of the PICC was 4.2 ± 2.3 hours of disc circumference (range: 1-10 hours) in width and 1363 ± 384μm in length (range: 732 - 2165μm). The mean thickness of the choroid (defined as the distance between Bruch´s membrane and the inner sclera surface) at the site of the PICC was 295 ± 79μm (range205-468μm). The most common location of the PICC was the inferior region (40%), followed by the nasal region (27%), the nasal superior region (13%), the nasal inferior region (13%), and finally the temporal superior region (7%). On fundus photos, a typical yellow-orange lesion was found in 8 out of the 15 (53%) eyes with PICCs on the OCT images. The other 7 eyes (47%) did not show any detected special ophthalmoscopical abnormality at the PICC location on the fundus photographs. 

The mean age of the participants with PICC was 67.2 ± 7.4 (range: 50-77), and 60% of them were men. The mean spherical equivalent, axial length, and intraocular pressure of the eyes with PICC was -9.7 ± 5.1 diopters (range -3.9 to -22.0 diopters), 28.0 ± 1.3 mm (range 26.7 to 30.69 mm), and 15.7 ± 2.5 mmHg (range: 11 - 20 mmHg), respectively. 

In univariate analysis, the presence of PICC was significantly associated with a lower optic disc tilted index (indicating a more marked disc tilting) (*P*<0.001), a more myopic refractive error (*P*<0.001), longer axial length (*P*<0.001), lower best corrected visual acuity (*P*<0.001), thinner subfoveal choroidal thickness (*P*<0.001), higher frequency of posterior staphylomata (*P*<0.001), higher frequency of photoreceptor inner segment / outer segment disruption (*P*<0.001), higher frequency of macular RPE / Bruch’s membrane complex disruption (*P*<0.001), presence of parapapillary gamma zone (*P*<0.001), presence of macular schisis (*P*=0.001), deeper anterior chamber depth (*P*=0.009), steeper corneal curvature (*P*=0.03), and presence of choroidal neovascularization / RPE clumping (*P*=0.04). It was not significantly associated with the systemic parameters of age (*P*=0.43), gender (*P*=0.20), body height (*P*=0.11), body weight (*P*=0.28), systolic blood pressure (*P*=0.55), diastolic blood pressure (*P*=0.92), cognitive function score (*P*=0.37), blood concentrations of glucose (*P*=0.61), HbA1c (*P*=0.20), creatinine (*P*=0.71), C-reactive protein (*P*=0.16), high-density lipoproteins (*P*=0.86), low-density lipoproteins (*P*=0.32), cholesterol (*P*=0.32) and triglycerides (*P*=0.84); nor with the ocular parameters of central corneal thickness (*P*=0.22), lens thickness (*P*=0.92), corneal diameter (*P*=0.16), cylindrical refractive error (*P*=0.07) and intraocular pressure (*P*=0.20). 

In the binary logistic regression analysis including the presence of PICC as dependent parameter and all those variables as independent parameters which were significantly associated with PICC in the univariate analysis, only optic disc tilted index (*P*=0.04, OR: 0.00007; 95%CI: 0.000001, 0.55) and presence of posterior staphylomata (*P*=0.046, OR: 0.004; 95%CI: 0.0002, 0.91) remained significantly associated with PICC. The other parameters were not significantly associated with PICC, including presence of choroidal neovascularization / RPE clumping (*P*=1.00), macular RPE / Bruch’s membrane complex disruption (*P*=1.00), parapapillary gamma zone (*P*=1.00), subfoveal choroidal thickness (*P*=0.76), photoreceptor inner segment/ outer segment disruption (*P*=0.54), corneal curvature (*P*=0.27), macular schisis (*P*=0.24), anterior chamber depth (*P*=0.20), refractive error (*P*=0.14), axial length (*P*=0.12) and best corrected visual acuity (*P*=0.11). 

## Discussion

In our population-based study applying enhanced depth imaging by spectral-domain OCT, PICCs were found only in highly myopic eyes with a prevalence of 16.9 ± 4.0%. The PICC were located most frequently at the inferior optic disc region. Half of the PICCs as found in OCT images showed a yellow-orange lesion on fundus photos suggesting that ophthalmoscopy alone may uncover only 50% of PICCs. In binary logistic regression analysis, presence of PICC was significantly associated only with a higher degree of optic disc tilting and the presence of posterior staphylomata. After adjusting for optic disc tilt and staphylomata, presence of PICCs was statistically independent of age, gender, body height, weight and body mass index, arterial blood pressure, diabetes mellitus, biochemical blood examinations, central corneal thickness, ocular biometric measure including axial length, refractive error, subfoveal choroidal thickness, parapapillary gamma zone and intraocular pressure. 

 Our study confirms other investigations in which PICCs were strongly associated with high myopia. In our study, PICCs were detected only in highly myopic eyes. Performing a multivariate analysis however, further elucidated, that PICCs were primarily not related to high myopia or abnormal long axial length but to a tilted optic disc shape and the presence of staphylomata. Since both features, disc tilting and staphylomata, usually occur in highly myopic eyes, the univariate analysis had suggested an association between high myopia and PICCs. In reality however, the distortion of the posterior fundus found in many but not all highly myopic eyes and associated with disc tilting and staphylomata may be the primary causative factor for the presence of PICCs in highly myopic eyes. The prevalence of PICCs in the highly myopic study population was markedly higher in our study than in the two previous hospital-based investigations (16.9% in our study versus 4.9% and 11.0% previously) [4,5]. The reason for the discrepancy may be differences in the study populations, in the study design (population-based versus hospital-based), in the ethnicity (Chinese versus Japanese) or others. In the study by Yeh and colleagues, PICCs were described to be also present in emmetropic and hyperopic eyes [10], while in our investigation, PICCs were detected only in highly myopic eyes. The reason for the discrepancy between both studies may have been differences in the definition of PICCs. In our study, a tube-like or round area with low reflection on the OCT images corresponding to a choroidal vessel on the fundus photographs was not considered to be a PICC but a large choroidal vessel (Figure 2). In Yeh´s study, the diagnosis of a PCC was “based on the OCT finding of an intrachoroidal hyporeflective space located below the normal plane of the RPE adjacent to the optic nerve" [10]. It could have led to the inclusion of some non-highly myopic eyes with large choroidal vessels into the study group of eyes with PICCs. Another difference between both studies was the device used: Yeh and colleagues performed their examinations with the OCT “Stratus” (Humphrey-Zeiss, Dublin, CA, U.S.A.) while we used the OCT Spectralis (Heidelberg Engineering Co., Heidelberg, Germany). 

 The existence of a cavitation or cleft between Bruch´s membrane as basal membrane of the RPE and the choriocapillaris and the choroid with Sattler´s and Haller´s layers of the medium and large choroidal vessels suggests that Bruch´s membrane is not firmly attached to the sclera by a strong choroidal adhesion. It supports the “sliding Bruch´s membrane theory” which suggests a temporal shift of Bruch´s membrane opening as the inner part of the optic nerve head in relationship to the scleral opening as the pouter part of the optic nerve head. Such a shift can explain an overhanging of Bruch´s membrane into the nasal optic disc and the development of a parapapillary gamma zone in the temporal region without Bruch´s membrane in medium or highly myopic eyes [16,20]. The sliding Bruch´s membrane theory has also been supported by observation of a marked diminution of parapapillary regions (presumably gamma zone) after a marked reduction in intraocular pressure [21]. The sliding Bruch´s membrane theory may also be of potential interest for the discussion on the development of high myopia. 

 Since the spatial resolution of enhanced depth imaging by OCT is not sufficient to make out the choriocapillaris on the outer surface of Bruch´s membrane, the question arises, whether the cleft in the choroid (leading to the choroidal cavitation) took place between Bruch´s membrane and the choriocapillaris or between the choriocapillaris and the remaining choroid. Assuming a generally strong adhesion between a structure such as the choriocapillaris and its basal membrane, one may assume that a PICC or choroidal cavitation is histologically an intrachoroidal cleft. If thus the choriocapillaris is still attached to Bruch´s membrane, the deep retinal layer would be better nourished than as if a PICC would be the correlate of a detachment of Bruch´s membrane from the choriocapillaris. The alternative is that a PICC represents a suprachoroidal cleft as also shown by Spaide and colleagues in some eyes [9]. Due to the limited special resolution of choroidal imaging it may have been up to now too difficult to clearly differentiate between suprachoroidal clefts and intrachoroidal fissures. Future histologic investigations or future clinical studies with even more advanced imaging techniques may shed some light onto that question. 

Potential limitations of our study should be mentioned. First, as for any population-based study, the rate of non-participation or non-availability of examination results can matter. In our study, the participation rate of all eligible subjects was 78.8%, which was comparable with other large population-based investigations. Readable OCT images of the optic disc were available for 94.5% of the whole population and 94.7% of the participants with highly myopia. Second, since fluorescein angiograms and indocyanine green angiograms were not available, the characteristics of the lesion on angiograms and the potential associations between blood flow and the PICC could not be evaluated. Third, only the right eyes of the study participants underwent enhanced depth imaging by spectral-domain OCT, so that the study design did not allow to assess inter-eye differences in the prevalence, size, location and associations of PICCs . 

 In conclusion, our population-based study on adult Chinese revealed a prevalence of PICCs of 16.9 ± 4.0% in the highly myopic group, while PICCs were not detected in non-highly myopic eyes. The PICCs were located most frequently inferior to the optic disc. Only half of the PICCs detected on OCT images showed a yellow-orange lesion on fundus photos. In multivariable regression analysis, presence of PICC was significantly associated only with a more pronounced optic disc tilting and presence of posterior staphylomata, while it was not associated axial length, refractive error or other ocular or systemic parameters. 
